# T6SS in plant pathogens: unique mechanisms in complex hosts

**DOI:** 10.1128/iai.00500-23

**Published:** 2024-08-21

**Authors:** Lexie M. Matte, Abigail V. Genal, Emily F. Landolt, Elizabeth S. Danka

**Affiliations:** 1 Biology Discipline, Division of Natural and Social Sciences, St. Norbert College, De Pere, Wisconsin, USA; Department of Microbiology and Environmental Toxicology, University of California at Santa Cruz, Santa Cruz, California, USA

**Keywords:** Type VI secretion systems, T6SS, effector proteins, phytopathogen, host-pathogen interactions, pathogenesis

## Abstract

Type VI secretion systems (T6SSs) are complex molecular machines that allow bacteria to deliver toxic effector proteins to neighboring bacterial and eukaryotic cells. Although initial work focused on the T6SS as a virulence mechanism of human pathogens, the field shifted to examine the use of T6SSs for interbacterial competition in various environments, including in the plant rhizosphere. Genes encoding the T6SS are estimated to be found in a quarter of all Gram-negative bacteria and are especially highly represented in *Proteobacteria*, a group which includes the most important bacterial phytopathogens. Many of these pathogens encode multiple distinct T6SS gene clusters which can include the core components of the apparatus as well as effector proteins. The T6SS is deployed by pathogens at multiple points as they colonize their hosts and establish an infection. In this review, we describe what is known about the use of T6SS by phytopathogens against plant hosts and non-plant organisms, keeping in mind that the structure of plants requires unique mechanisms of attack that are distinct from the mechanisms used for interbacterial interactions and against animal hosts. While the interactions of specific effectors (such as phospholipases, endonucleases, peptidases, and amidases) with targets have been well described in the context of interbacterial competition and in some eukaryotic interactions, this review highlights the need for future studies to assess the activity of phytobacterial T6SS effectors against plant cells.

## INTRODUCTION

### Overview of plant structures and the locations of associated bacteria

Plants are complex, eukaryotic organisms with specialized tissues and structures that play important agricultural, medicinal, structural, and recreational uses for humans. Individual plant cells contain membrane-bound organelles, including a nucleus, and are protected by a layered cell wall primarily made of the polysaccharide cellulose and glycoproteins (1). Although there is a wide variation in plant structure, most plants have roots that are embedded in a substrate and anchor the plant in place (2). A supportive stem grows from the roots, and outgrowths of leaves sprout from the stem. In addition to anchoring the plant, the roots will collect water and nutrients from the soil. Stems contain the vasculature responsible for transporting materials between the roots and the rest of the plant. The xylem and intracellular connections known as plasmodesmata are primarily responsible for transporting water and minerals from the roots to the rest of the plant, while the phloem transports organic molecules like sugars, hormones, and amino acids from the shoots of the plant down to the roots. This organization means that plant cells and tissues are highly interconnected and are susceptible to disruption and invasion by bacterial pathogens.

Bacteria can be associated with the surfaces of all parts of plants, including roots, stems, leaves, and reproductive structures (flowers, fruits, and seeds), and less commonly, within the plant as endophytes (3). Endophytes can be found living within host cells, in the vasculature, or outside of cells but within the tissue (the apoplast). Some endophytes live within a unique plant-based structure called a nodule on the roots of the plant. The process of nodulation involves a complex interplay of bacterial and plant signals which promotes the development of the root nodule structure and the stable integration of the bacteria (2). External or epiphytic bacteria are commonly found on the surfaces of leaves (phyllobacteria) and in dense communities on the roots of plants (rhizobacteria) (3). Both phyllobacteria and rhizobacteria can receive secreted nutrients and signals from the plant and can also produce chemicals that affect plant growth and behavior. Importantly, the organisms that make up the plant microbiota can also be involved in interbacterial interactions that kill potential plant pathogens to protect the plant or can be opportunistic pathogens that cause damage to the plant. Infection can occur when organisms gain access to the interior of the plant and begin to proliferate. These plant pathogens (phytopathogens) encode many molecular tools that promote pathogenesis, including, but not limited to, the Type VI secretion system (T6SS).

### The organization of Type VI secretion systems (T6SSs)

**Fig 1:**
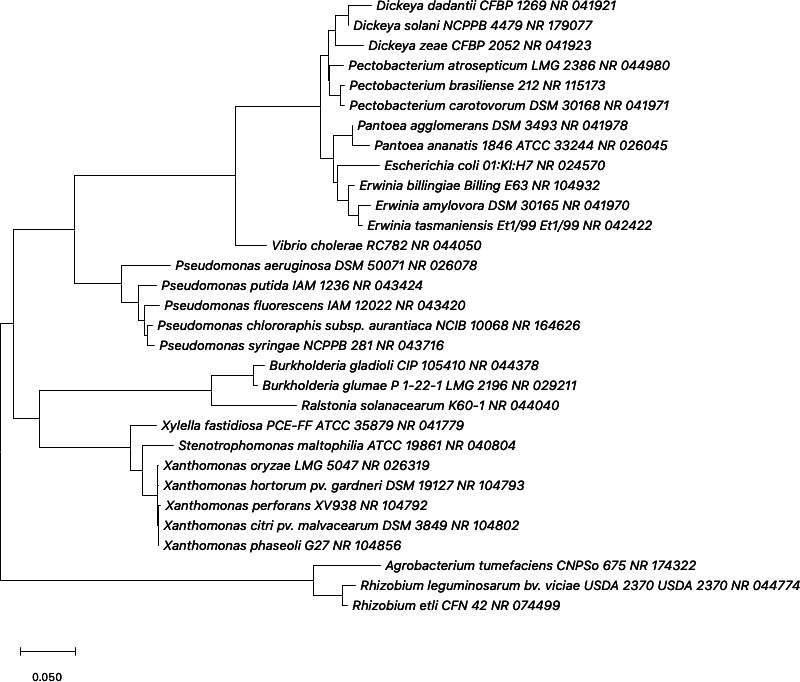
Phylogenetic relationships of key T6SS+ phytopathogens. An unrooted maximum likelihood tree was generated in MEGA11 using 16S rRNA gene sequences for the indicated organisms. The evolutionary history was inferred over 500 bootstrap replicates using the general time reversible model with a discrete gamma distribution to model evolutionary rate differences among sites (+*G*). The rate variation model allowed for some sites to be evolutionarily invariable (+*I*). The tree is drawn to scale, with branch lengths measured in the number of substitutions per site. The organism names, strain names, and accession numbers are included on each branch.

Bacteria use secretion systems to move proteins out of the cytoplasm and into membranes, the periplasm, the extracellular environment, and even neighboring cells. Currently, secretion systems ranging from Type I to Type XI have been proposed as distinct mechanisms, with some making the case for outer membrane vesicles to represent Type 0 secretion systems (4–6). Secreted proteins extend the range of bacterial cell interactions and can be particularly impactful in the context of infection. In this review, we focus on the dynamic mechanism of protein secretion known as the T6SS. The genes encoding this system were first identified in the plant pathogen *Rhizobium leguminosarum*, with the name Type VI secretion being assigned 3 years later to conserved genes identified in *Vibrio cholerae* (7, 8). These genes were initially thought to be used for virulence in animal systems and then were determined to simultaneously mediate interbacterial interactions (9–11). The field has exploded from there. T6SSs are widely distributed across Gram-negative bacteria, including genera in the phyla *Acidobacteria*, *Bacteroidetes*, *Deferribacteres*, *Gemmatimonadetes*, *Nitrospirae*, *Planctomycetes*, and *Proteobacteria* (12, 13). These systems are especially abundant in *Proteobacteria*, with complete gene clusters frequently found on the chromosomes of γ-*Proteobacteria* and on plasmids in ɑ- and β-*Proteobacteria* (13, 14). Numerous pathogens of plants and animals are classified in these groups. Recent work has investigated the use of T6SSs as protective mechanisms within the rhizosphere, and the field is continuing to explore T6SS in bacterial plant pathogens (15). This review focuses on phytopathogens in approximately a dozen genera, representing ɑ-, β-, and γ-*Proteobacteria*. A phylogenetic tree demonstrating the relatedness between these organisms is shown in Fig. 1.

**Fig 2:**
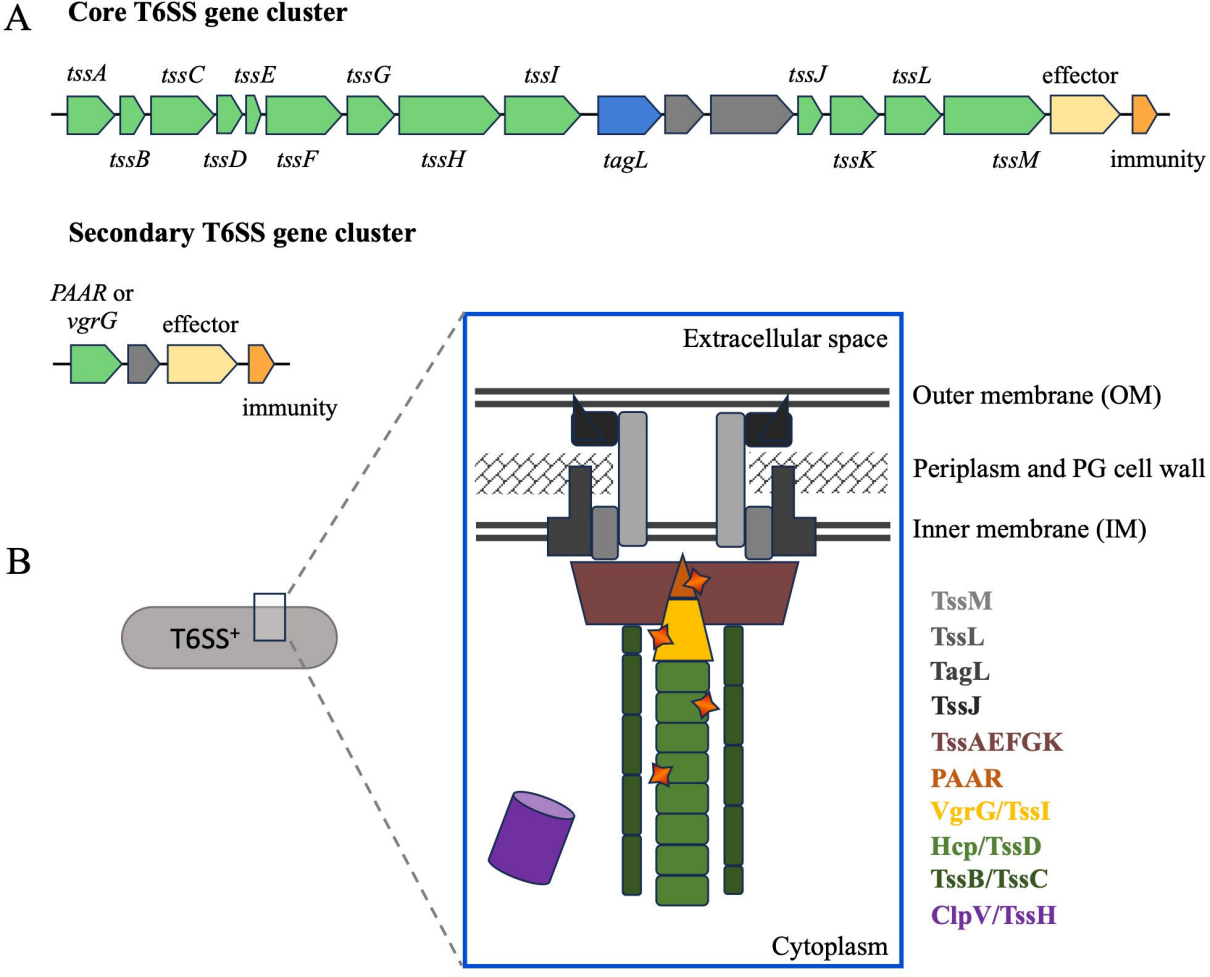
Organization of T6SS genes and the T6SS apparatus. (**A**) The simplest core T6SS gene clusters contain the 13 genes required to build the membrane apparatus (green) but may also include accessory genes (blue), effector and immunity pairs (yellow and orange), or unrelated interspersed genes (gray) (top). Separate T6SS gene clusters usually contain only a few genes, such as a *PAAR* or *vgrG*, along with an accessory gene and an effector and immunity pair (middle). (**B**) The protein apparatus assembles in the cytoplasm and cell envelope of cells. Proteins TssLJM and TagL anchor the protein in the membranes and cell wall; TssAEFGK forms the baseplate that connects the TssB/C outer sheath to the membrane, and the inner tube is composed of Hcp/TssI proteins and topped with VgrG/TssI and a PAAR protein. Effector proteins are shown as stars on Hcp, VgrG, and PAAR. The cytoplasmic ClpV ATPase associates with the outer sheath to promote the disassembly of the apparatus after the injection mechanism has been triggered.

In their simplest form, T6SS gene clusters contain the 13 core genes, *tssA-tssM*, that are required to build the complex injection system (Fig. 2A) (8, 16, 17). When assembled inside the cell, this structure has an inner tube surrounded by an outer sheath which is anchored in the bacterial cell envelope by the TssJ, TssL, and TssM proteins (Fig. 2B) (10, 18, 19). These proteins associate with the inner membrane (TssL and TssM) and the outer membrane (TssJ lipoprotein) to hold the structure in place. In many organisms, the peptidoglycan-binding domain of an accessory protein known as TagL (Tag for tss-associated gene) provides the physical connection between the envelope anchoring proteins and the cell wall (10, 18). A cytoplasmic baseplate composed of TssA, TssE, TssF, TssG, and TssK attaches the outer sheath to the envelope anchoring proteins (20). The outer sheath is made of a helix of TssB and TssC proteins (21). Within the outer sheath, stacked Hcp (TssD) proteins form an inner tube that is capped by a trimer of VgrG (TssI) proteins (9, 22). The VgrG cap is often topped with a spike-like PAAR protein (23). The final protein in the core structure is the *tssH*-encoded ClpV enzyme. This ATPase facilitates the disassembly of the outer sheath inside the donor cell so that the protein subunits can be reused after the inner tube has been deployed (21).

The assembled structure uses a phage- or syringe-like mechanism to deliver core components or associated effector proteins from the cytoplasm of the donor cell into neighboring cells in one step (24, 25). This is accomplished by contracting the proteins that make up the outer sheath so that the inner tube is extended through the outer membrane of the donor cell and into the target cell (21, 26). Hcp and VgrG proteins can impact the attacked target cell on their own, but the delivery of toxic effector proteins is most frequently the cause of death in the receiving cell (27, 28). These effector proteins are often encoded outside of the core T6SS gene cluster. While some organisms only encode one T6SS effector, other organisms contain multiple genes (some include a dozen or more) that are predicted to encode effectors (7, 29–33). The effector proteins can be associated with the Hcp proteins that make up the inner conduit or with the VgrG or PAAR proteins that comprise the tip (23, 34–36). As the apparatus is injected into the target cell, the associated effector proteins are carried along and delivered to the target. Organisms frequently encode multiple Hcp, VgrG, and PAAR proteins that may be used to deliver specific effectors in particular circumstances (37, 38). Together, the variability of VgrG, PAAR, and effector proteins indicates that T6SSs have interchangeable components that may contribute to the use of these systems in many organisms and for multiple, distinct purposes.

Bacteria with T6SSs encode genes for immunity proteins that prevent autotoxicity from the effector proteins before they are delivered and will prevent killing of bacterial kin (39–41). However, these immunity proteins may not be necessary if the effector target is a unique structure in a plant cell. Effector and immunity genes can be associated with the core T6SS gene cluster or at separate loci throughout the genome (27, 39, 42). When these pairs are not within a core cluster, the effector-immunity genes are usually contained in operons that also encode a VgrG, Hcp, PAAR protein, or accessory adaptor protein that can help with the identification and association of the effector protein with the core structure (Fig. 2A) (38, 43–45). These accessory proteins are not found in all organisms but may aid in loading VgrG and effector proteins onto the Hcp tube. Many of the accessory proteins that have been identified so far include homologous, but uncharacterized, domains such as DUF4123, DUF1795, or DUF2169 (38, 44, 46). In this review, we explore the use of T6SS in plant pathogens.

## PLANTS PRESENT UNIQUE TARGETS FOR BACTERIAL T6SSs

**Fig 3:**
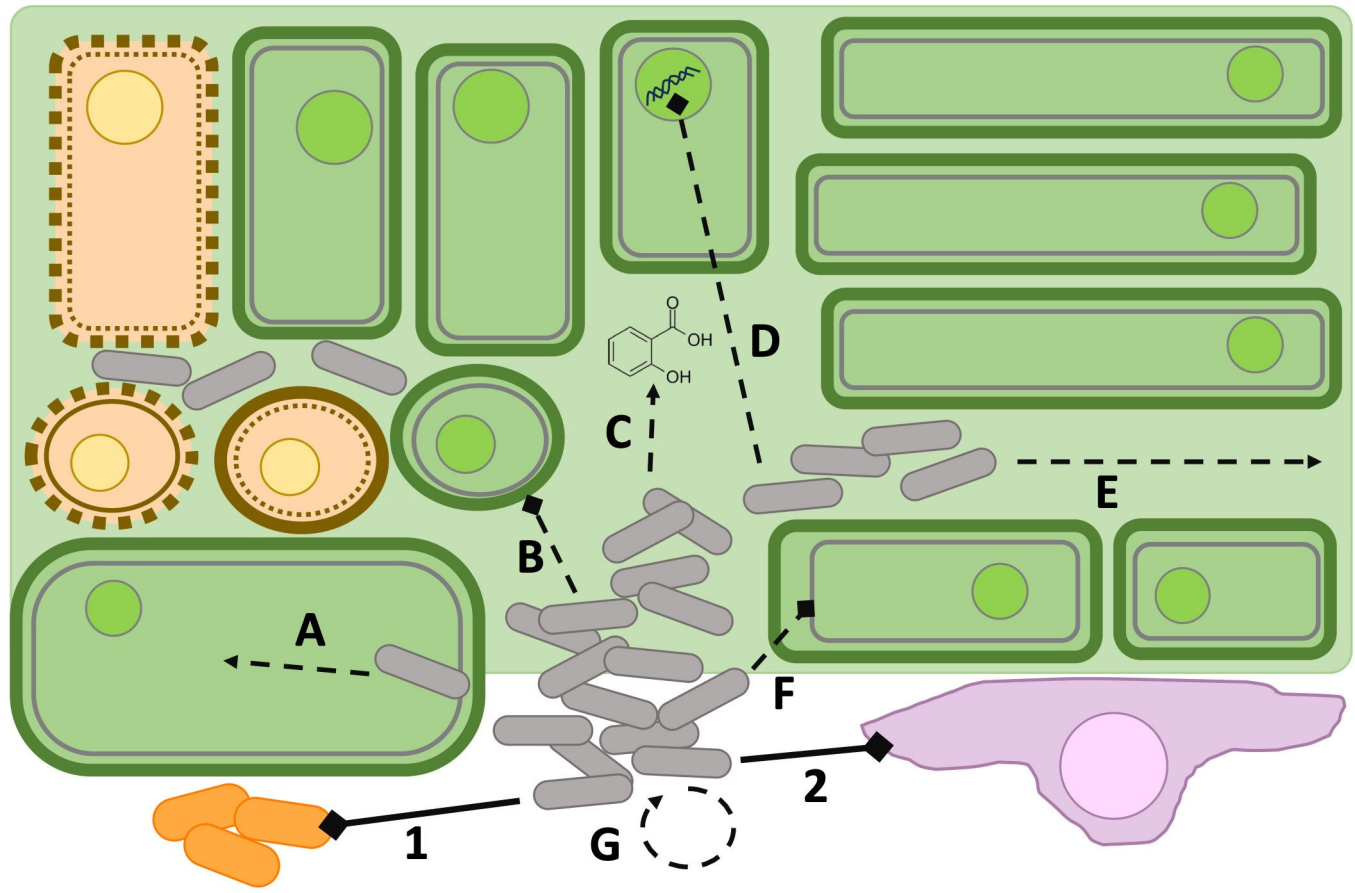
Roles of the phytopathogen T6SS during infection. T6SSs of phytopathogens can be used against both non-plant (1 and 2) and plant targets (A–G). Outside of the plant, T6SS can kill other plant-associated bacteria (1, orange bacilli; bottom left) and defend T6SS+ cells against predation by amoeba (2, lavender irregular eukaryotic cell; bottom right). These non-plant targets are shown with solid lines and numbers. Within plant hosts, T6SS can impact nodulation (A), plant cell wall integrity (B), host compatibility and/or defense responses in plants (C), host nucleic acids (D), colonization of plant tissues and/or spread away from the initial site of infection (E), plant cell membrane integrity (F), or the regulation of other virulence factors such as motility, other secretion systems, and exopolysaccharide production (G). The use of T6SS may result in plant cell death due to cell wall lysis or membrane disruption, or a combination of both (brown eukaryotic cells, top left). Plant targets are shown with dashed lines and letters. Thin gray lines represent plant cell membranes and thick green lines represent plant cell walls.

T6SS effector proteins must impact essential structures or molecules in target cells to kill those cells. Effector proteins that compromise the integrity of the cell membrane (phospholipases) or peptidoglycan cell wall (amidases, glycosidases, and muramidases) or that degrade DNA (nucleases) are particularly effectual (40, 43, 47, 48). Cell membranes can also be targeted in animal eukaryotic cells, along with the critical structural molecule actin (49, 50). Although the cell membranes of plant eukaryotic cells may be important targets, plant structural organization presents unique challenges. We describe what is known and hypothesized about the mechanisms by which bacterial phytopathogens use T6SSs to directly impact their plant hosts and cause disease (Fig. 3A through G).

### Phytopathogens use enzymes to break down plant cell walls

The plant cell wall is an essential cellular component that allows for the aerial structure of plants as well as the expansive burrowing action of plant roots (2). While cell walls in bacteria are formed of peptidoglycan, plant cell walls contain the polysaccharides cellulose, hemicellulose, and pectin, along with lignin and glycoproteins. Both peptidoglycan and cellulose contain ß-1,4-glycosidic bonds connecting sugars, but the differing monosaccharides in the chains (glucose in cellulose vs *N*-acetylglucosamine and *N*-acetylmuramic acid in peptidoglycan) make it unlikely that the same enzyme can cleave both polysaccharides (1). Instead, plant cell wall targeting will require enzymes different from those that can alter the bacterial cell wall and provides a unique opportunity for organisms to produce an effector with a function that is specific to the target cell and cannot affect the donor cell. This highlights the specificity of some T6SS effectors and reiterates the idea that phytopathogens need to encode effectors that are specific to their targeted hosts.

The plant cell wall is also a formidable structure for bacteria to contend with, as the cell walls can vary in width from approximately 0.1 to 10.0 µm (51). It is important to note that the organization of the cell wall and the cell membrane of plant cells is more similar to the envelope structure of Gram-positive bacteria as opposed to the outer membrane-covered cell wall found in Gram-negative bacteria. This structural organization could make the plant cell wall more susceptible to attack by bacterial phytopathogens. The exposed cell wall of Gram-positive bacteria can be targeted by a peptidoglycan hydrolase effector of Gram-negative organisms in a contact-dependent manner, and a similar mechanism could function in plant cells (52). In terms of scale, plant cell walls are thicker than the cell walls of Gram-positive cells, but the thinnest plant cell walls are approximately the same thickness as fungal cell walls, and T6SS effectors can be delivered across fungal cell walls (53, 54). This suggests that the plant cell wall could be a useful target for the T6SS of phytopathogens.

Bacterial pathogens responsible for soft rot diseases in plants are known to utilize a variety of plant cell wall-degrading enzymes (PCWDEs) that result in the characteristic tissue maceration of soft rot diseases (55). Several phytopathogens encode predicted effectors with homology to pectinases and other potential plant cell wall-degrading enzymes. These enzymes are highly represented in the genomes of *Dickeya dadantii* and *Dickeya solani* and are predicted to delivered via T6SS (56). An *in silico* analysis of the T6SS secretome of *Pectobacterium brasiliense* identified several enzymes including pectate lyases, glycosyl hydrolases, pectin esterases, and endoglucanases that would likely degrade plant cell walls (57). An additional analysis of seven *Pectobacterium* spp. identified cellulases and a variety of pectin-degrading enzymes as potential T6SS effectors that were found across most strains (58). While the activity of the PCWDEs is known to be important for full virulence, whether these enzymes can be delivered via T6SS needs to be elucidated (Fig. 3B). Some of the cellulases, hemicellulases, pectinases, and proteases produced by *Burkholderia* spp., *Dickeya dadantii*, *Erwinia amylovora*, *Pectobacterium* spp., *Ralstonia solanacereum*, *Xanthomonas* spp., and *Xylella fastidiosa* are known to be important effectors of T2SS (59). A portion of these T2SS effectors are predicted to also be substrates for T6SS, opening the door for interesting crosstalk between the two secretion systems (60).

Finally, an intact T6SS allows the phytopathogen *Pseudomonas syringae* to outcompete the yeast *Cryptococcus carnescens* (61). Both copies of *hcp* encoded by *P. syringae* must be expressed for the bacteria to maximally inhibit the growth of *C. carnescens* in mixed culture. The growth inhibition of yeast may be due to targeting of the yeast cell wall. As *P. syringae* is likely to encounter yeasts in the microbiota of plants, this mechanism may help the bacteria colonize the plant before causing infection.

### Plant cell membranes are putative T6SS targets

T6SS effectors with lipase and phospholipase activity are known to be effective against both bacterial and eukaryotic cells, as demonstrated by experiments utilizing mammalian cell culture lines and amoeba (47, 49, 62, 63). Plant cells also contain the targets of these enzymes in their cell and organelle membranes (Fig. 3F). It is therefore not surprising that many different phytopathogens including *Burkholderia* spp., *Erwinia tasmaniensis* and *Erwinia billingiae*, *Pectobacterium* spp., *Pseudomonas chlororaphis*, and all T6SS+ species within the *Ralstonia solanacearum* species complex encode putative T6SS effectors with homology to lipases or phospholipases (33, 57, 58, 64–66). In addition, a survey of T6SS gene clusters in bacterial plasmids determined that nearly 80% of *Ralstonia solanacearum* plasmids harbor putative lipase effectors (14). It is important to note that the biochemical activity has not yet been confirmed for any of these putative effectors. The mechanism by which the effectors are delivered to the plant cells will also need to be investigated, keeping in mind the barrier posed by the plant cell wall. Confirmatory experiments could be modeled off prior work focused on T2SS, which confirmed roles for lipase effectors in *Burkholderia* spp., *Pseudomonas* spp., *Xanthomonas* spp., and *Xylella fastidiosa* (59).

### Effector proteins modify host cell nucleic acids

DNA and RNA in bacteria are common targets of T6SS nuclease-containing effector proteins, and it is likely that plant nucleic acids can also be targeted by the T6SS of phytopathogens (Fig. 3D). Indeed, *in silico* analyses of phytopathogen genomes have identified putative T6SS nucleases. A pangenomic analysis of *Ralstonia* spp. identified multiple auxiliary effector/immunity clusters that were predicted to encode nuclease effectors (14, 33). The T6SS secretome of *Pectobacterium* spp. is predicted to include endonucleases and a VRR-Nuc domain-containing protein (57, 58). In T6SS-encoding *Salmonella* spp., proteins with VRR-Nuc domains cleave and inactivate nucleic acids when single-stranded structures are formed (67). Phytopathogen *Pseudomonas syringae* produces an Rhs protein that undergoes self-cleavage to produce three distinct fragments, of which the C-terminus has endonuclease activity (68). This endonuclease is delivered via the T6SS and will kill *Escherichia coli* unless the downstream immunity protein is expressed in the *E*. *coli*, but the activity of this effector has not yet been tested in a plant host.

A separate mechanism of host cell nucleic acid modification is used by the T4SS of *Agrobacterium tumefaciens*. T4SS delivers T-DNA, for example, the Ti-plasmid, or protein effectors to plant host cells (69). In *Agrobacterium*, the effectors seem to be important for the translocation of DNA into and through the host cells and may independently affect the survival and activity of the cells (70–72).

### T6SS expression can be co-regulated with other virulence factors

T6SS expression is often coordinated with the expression of other virulence factors, including secretion systems and motility factors (Fig. 3G). In *Agrobacterium tumefaciens*, the T4SS and T6SS systems are inversely expressed such that the T6SS is upregulated when the cells are first exposed to the rhizosphere but is downregulated during virulence (73–77). In *Agrobacterium*, the T6SS is used to establish a niche, and then the expression of this secretion system is downregulated to allow for the expression of the T4SS that delivers molecules used to develop infection. The T6SS is also linked to the T4SS in *Pectobacterium* spp. as an estimated 16% of the proteins secreted by the T6SS in *P. brasiliense* may also be secreted by other secretion systems, including T4SS (57). Additional virulence factors, including T3SS, are co-regulated with *E. amylovora* T6SS gene clusters (78). Mutation of core T6SS genes induced genes for flagella synthesis, chemotaxis, and one T3SS, while genes for a second T3SS and iron acquisition were downregulated. Swimming motility is also affected in T6SS mutants of *Xanthomonas phaseoli*, although the motility was decreased in a *clpV* mutant (79). Swimming motility and biofilm formation are important as *Xanthomonas* spp. enter deeper plant tissues and cause infection (80). Finally, secretome studies comparing *Pseudomonas syringae* strains with high and low virulence found that strains with higher virulence more commonly secreted T6SS core proteins (Hcp and VgrG), proteins that may represent novel T6SS effectors (including serralysin, alkaline metalloendoprotease, YD repeat protein, TonB-dependent siderophore receptor, and hemolysin-type calcium-binding peptidase), and flagellar proteins (81). In contrast, T3SS effector proteins were found at lower levels in the supernatants of these higher virulence strains and highly expressed in the less virulent strains. Together, these data demonstrate that complex regulatory mechanisms control the expression of T6SSs, along with other critical virulence factors.

### T6SS can alter host compatibility and promote nodulation

The first report of the T6SS was described in *Rhizobium leguminosarum*, although the system was not named as a T6SS for a few years (7). The authors noted that nodulation was disrupted in the mutant of interest because the mutant was unable to establish the usual symbiotic relationship with *Pisum sativum* (Fig. 3A) (7). The complex process of nodulation uses T3SS, T4SS, and T6SS and may represent a modification of former pathogenic systems that were used to suppress the plant response to infection (7, 82–84). The authors hypothesized that protein secretion triggers a hypersensitive response in plants that are not natural hosts for *R. leguminosarum*, leading to a stunted phenotype (7). Importantly, the authors showed that cell-free *R. leguminosarum* culture supernatant affected nodulation, indicating that the T6SS effectors do not need to be translocated into the plant cells to have an effect.

More recently, the T6SS of *Rhizobium etli* was examined in the context of nodulation and bacterial competition (85, 86). The T6SS is required for *R. etli* to successfully establish a relationship with its native plant host, *Phaseolus vulgaris*, and may be used to determine host compatibility (Fig. 3C) (85). T6SS gene expression is upregulated in the presence of native hosts, after exposure to root exudate, and when the cells are established within root nodules. When bacteria without a functional T6SS are exposed to their plant hosts, the root nodules form less frequently and are smaller, and the plants produce less mass than plants that are colonized with a T6SS-proficient strain. Before nodulation can begin, *Rhizobium etli* must first colonize plant roots. A putative effector-immunity gene pair downstream of the core T6SS cluster does not affect the nodulation capability of *R. etli* (86). However, the effector protein can kill *E. coli* cells, and *R. etli* cells that do not produce the effector protein are less competitive in colonizing plant roots. This suggests that *Rhizobium* spp. use T6SS at multiple points during colonization and nodulation of roots.

### T6SS promotes colonization of plant tissues and spread away from the initial site of infection

The environmental conditions within leaves and stems present new challenges for pathogens as they transition away from an epiphytic stage. Increased water and nutrient availability, coupled with plant signaling factors and plant defense factors, often lead to changes in bacterial gene expression including T6SS genes. When *Pectobacterium atrosepticum* and *Pectobacterium carotovorum* are grown in media containing plant extracts *in vitro* or in potato tubers, expression of core T6SS genes is quickly upregulated (87–89). *Dickeya dandantii* similarly upregulates T6SS expression as the bacterial cells move from the surface of *Arabidopsis thailiana* leaves into plant tissues, suggesting that T6SS is required for adaptation to this new environment (60).

Once inside the plant, a functional T6SS is important for many pathogens as they grow and spread within tissues to cause disease (Fig. 3E). This was demonstrated using infection models for *Erwinia amylovora*, multiple *Xanthomonas* spp., *Pantoea ananatis*, *Burkholderia glumae*, and *Ralstonia solanareceum. Erwinia amylovora* T6SS mutant strains had decreased virulence and developed smaller lesions when inoculated onto apple flowers or the surface of unripe pear fruits (78, 90). Interestingly, the T6SS mutant strains caused larger wounds in apple stems over time, which the authors attribute to decreased production of external factors that normally encourage bacterial adherence within the stems, but increased spread when these factors are scarce (78). Decreased production of the polysaccharides amylovoran and levan in T6SS mutants could affect the production of bacterial exopolysaccharide and disease progression in plant hosts (90). *Pantoea ananatis* encodes two T6SS clusters, one that contains all of the core components and one truncated cluster with two core genes and four accessory proteins (91). When the complete core component-containing cluster is mutated, *P. anantis* virulence in onion plants is limited, but this virulence defect is not observed when the truncated T6SS cluster with accessory proteins is inactivated. However, the DUF2169 domain-containing accessory protein is important for virulence in *Burkholderia glumae* (92). In this organism, two of the four encoded T6SS gene clusters that contain core components directly affect the progression of disease in rice stems and on rice panicles, while the other two clusters are dispensable (93). *B. glumae* also encodes two copies of a DUF2169 domain-containing protein, one of which is required for virulence (92). The authors hypothesize that the bacterial cells are unable to deliver effectors to host cells without this protein. Decreased virulence was observed when *Ralstonia solanacereum tssM*, *ttsB*, *hcp*, and *vgrG* mutants were tested in tomato and eggplant models of infection using both soil drench inoculation and petiole inoculation (94–96). Strains deficient in T6SS did not produce the same levels of wilt as wild-type strains when tested in hosts (95, 96). *Ralstonia* causes bacterial wilt in plants when the cells form biofilms that block the flow of liquids in the xylem (97). Deletion of the *tssB* and *tssM* genes prevented the secretion of Hcp, decreased biofilm formation and cellular motility, and resulted in lower levels of root and stem colonization after soil inoculation (94, 95). Finally, disrupting the T6SS in *Xanthomonas oryzae*, *Xanthomonas perforans*, and *X. phaseoli* affects the virulence of these organisms in rice, tomato, and cassava hosts, respectively (79, 98, 99). It is unclear if these effects are due to core components, like Hcp and VgrG, or if effector proteins are being delivered by the T6SS. For example, when a core component of the T6SS, *tssM*, is disrupted in *X. perforans*, the mutant strain is more virulent in tomato plants, causing more serious disease with higher bacterial titers (98). Future studies can tease apart the roles of individual core and effector proteins during infection.

Phytopathogens also use effectors delivered by T3SS to interrupt the generation of a defensive response in a plant. For instance, *Pseudomonas syringae* T3SS effectors AvrPto and AvrPtoB block immune signaling, and AvrE effectors prevent the use of salicylic acid-mediated plant defenses and *Pseudomonas fluorescens* effectors RopAA, RopB, and RopM can suppress production of reactive oxygen species and the hypersensitive response (100–102). The immune suppression phenotype is not rare. One study found that over 90% of the encoded T3SS effectors were able to suppress the plant immune response to some degree (103). The delivery of T3SS effectors such as HopM1 and AvrE in *Pseudomonas syringae* and AvrHah1 in *Xanthomonas gardneri* increases water availability in the apoplast, generating the water soaking phenotype, which promotes nutrient availability for the pathogen and invasion into the tissue and may disrupt the plant immune response (104, 105). Future work on T6SS may explore whether these T3SS effectors can also be secreted through T6SS or whether T6SS-specific effectors can also modulate the host immune response.

### Seed to seedling transmission is enhanced by T6SS

*Xanthomonas perforans* is a seedborne pathogen that causes disease in tomato plants (98). After the seed germinates, *X. perforans* must first become established as an epiphyte within the phyllosphere before it can enter leaves through stomata or a wound. When a core component of the T6SS, *tssM*, is disrupted in *X. perforans*, the mutant has decreased epiphytic colonization capabilities and decreased transmission in high-humidity environments, such as those commonly found in commercial greenhouses. The *tssM* mutant strain also causes more severe disease than the wild-type strain during seed to seedling transmission. Finally, this mutant also has increased virulence in mature tomato plants, causing more severe disease with higher bacterial titers. These data together suggest that *X. perforans* T6SS is involved in early colonization and adaptation to the host. These interactions may also involve other microbes in the phyllosphere, but this was not directly tested. Later in infection, the *X. perforans* T6SS moderates the scale of the infection within the plant host.

## USES OF T6SS BY PHYTOPATHOGENS AGAINST NON-PLANT TARGETS

The rhizosphere and plant microbiota are populated with bacteria, fungi, amoeba, and viruses. Successful phytopathogens must be able to survive in this complex environment before they can infect the intended plant host. Therefore, in addition to directly targeting plant hosts, many phytopathogens also use T6SSs for interbacterial interactions and to kill predatory eukaryotic cells (Fig. 3). Both uses indirectly promote the development of disease in hosts when the targeted bacteria or amoeba normally acts to restrict the growth of potential pathogens.

### T6SS-mediated killing of plant-associated bacteria

It is well established that bacteria use T6SS to kill other bacteria in various niches. The ability of phytopathogens to use T6SS to kill bacterial competitors was also explored in many of the reports described above. These interbacterial interactions occur in both the phyllosphere and the rhizosphere, and while they often help the pathogens establish a foothold, some plant growth-promoting bacteria can also use T6SS to defend plants from potential attackers (Fig. 3, bottom left). Pathogens like *Agrobacterium tumefaciens* deploy T6SS to establish a niche during interbacterial competition in the microbe-rich rhizosphere (76, 106–108). Expression of the T6SS system is upregulated when the cells are first exposed to the rhizosphere, where the roles of DNase- and amidase-encoding bactericidal effectors in *A. tumefaciens* have been noted for their ability to inhibit the growth of other bacterial species (75, 76, 108, 109). The rice pathogen *Burkholderia gladioli* must also establish a niche in the plant microbiota before causing infection. Two T6SS effectors in *B. gladioli* have been characterized as nucleases and function to kill a range of bacteria (*E. coli*, *Agrobacterium tumefaciens*, and rice endophytes *Stenotrophomonas maltophilia* and *Sphingomonas* sp.) (110). *B. gladioli* also uses T3SS to break down and feed on fungi, further emphasizing the importance of secretion systems during competition and growth in the rhizosphere (111). Finally, although the T6SS of *Pectobacterium carotovorum* is not involved in interbacterial competition against other plant-associated bacteria *in vitro*, it allows T6SS+ cells to outcompete other bacteria when they are co-inoculated into a plant host (112). This indicates that while T6SS-mediated interbacterial competition is important on the surface of the plant before infection, these interbacterial interactions can continue as the bacteria invade plants.

Both commensal and pathogenic organisms encode and use T6SS. Organisms in the *Pantoea* genus are found in soil and water and can be associated with animal and plant hosts as epiphytes and pathogens (113). *Pantoea* spp. can promote the growth of rice, peppers, papaya, and potatoes through nitrogen fixation and the production of phytohormones and siderophores (114, 115). Select *Pantoea* spp. are used as biocontrol agents and may use the T6SS to limit the growth of other pathogenic bacteria and fungi (113, 116). However, pathogenic *Pantoea agglomerans* pv. *betae* and *P. ananatis* also employ T6SS. In *P. agglomerans* pv. *betae*, T6SS is used to outcompete other plant-associated bacteria and in intraspecies antagonistic interactions (116, 117). *P. ananatis* requires a complete T6SS gene cluster that contains all core components for interbacterial competition and for virulence in plants (91). The use of T6SS is also seen in plant growth-promoting bacteria in the genus *Pseudomonas. Pseudomonas* spp. are known for their ability to interact with a diverse range of hosts and for encoding multiple, powerful T6SSs that can obliterate other bacteria when provoked (118). *Pseudomonas putida* promotes plant growth and acts as a biocontrol agent that uses T6SS to kill phytopathogens, including *P. syringae*, *Xanthomonas campestris*, *Agrobacterium tumefaciens*, and *Pectobacterium carotovorum* (119). Similarly, plant growth-promoting *P. fluorescens* and *P. chlororaphis* encodes multiple T6SSs that are effective against bacteria and are required for the persistence of *P. chloraraphis* within the rhizosphere (66, 120). Finally, organisms in the phytopathogenic *P. syringae* complex use the T6SS to kill other bacteria, including those commonly associated with plant hosts, and to promote virulence in a variety of crops (61, 121, 122). Altogether, the T6SS is an important factor for both phytopathogens and plant growth-promoting bacteria as they fight to survive in the plant microbiota.

### Defense against predatory amoeba via T6SS

Phytopathogen *Xanthomonas citri* encodes two T6SS clusters (123). Regulation of these T6SSs is controlled at the level of transcription by environmental signals that upregulate expression of the gene clusters when the bacterial cells are on the surface of plants and downregulate expression as the bacteria move into the plant leaves (124, 125). Further, expression of the T6SS is triggered when *X. citri* cells are in the presence of the amoeba *Dictyostelium discoideum* for extended periods of time (123, 125). This differential regulation of T6SS expression results in functional differences. T6SS mutants were uniquely susceptible to predation by *D. discoideum* when compared to T2SS, T3SS, and T4SS mutants, even though the T3SS is also highly expressed when *X. citri* is on the surface of leaves (123, 124). Likewise, *Pseudomonas syringae* requires Hcp and an intact T6SS to resist predation by *Acanthamoeba polyphaga* (61). When both copies of the *P. syringae hcp* are deleted, the bacterial population is nearly eliminated over the course of seven days, even though the wild-type cells can partially resist predation. These data indicate that the T6SS represents an important defensive mechanism that promotes the persistence of phytopathogens in the plant microbiota before they cause disease (Fig. 3, bottom right).

Interestingly, the anti-amoeba role of T6SS is not unique to phytopathogens. The use of T6SS against predatory amoeba has also been shown in the plant growth-promoting bacterium *Pseudomonas chlororaphis*, which encodes two T6SSs (66). *D. discoideum* showed limited grazing and began to display aggregation phenotypes associated with starvation when wild-type *P. chlororaphis* or single T6SS mutants were provided as prey. However, a double T6SS mutant was unable to defend against the amoeba, as the bacterial cells were readily cleared by *D. discoideum* and the starvation phenotype was not observed. Therefore, the T6SS can be used by both phytopathogens and plant growth-promoting bacteria to defend themselves against predatory amoeba.

## REMAINING QUESTIONS AND A LOOK FORWARD FOR THE FIELD

T6SSs are widespread throughout Gram-negative organisms, including many phytopathogens, where they play important roles in plant colonization, nodulation, virulence and spread throughout a host, and transmission to other hosts. Of these roles, the molecular mechanisms of specific T6SS proteins have only been described for interbacterial competition. While the T6SS-associated phenotypes have been hypothesized to be caused by effector proteins, VgrG proteins, PAAR proteins, and/or Hcp proteins, it remains unclear whether individual T6SS proteins can degrade plant cell walls or plant cell membranes, modify host nucleic acids, alter plant immunity pathways, or induce plant cell death through other pathways (Fig. 3). *In silico* analyses can be used to identify and predict the functions of putative T6SS effectors, but experimental work in plants will be required to fully define the roles of these proteins. The development of new methods and models may be necessary to accurately characterize the targets of these proteins and to determine how those impacted targets affect the host and progression of infection. As examining the mechanisms of plant cell death and the immune responses in infected cells could provide clues about the roles and functions of T6SS effector proteins, collaborations with plant physiologists may prove particularly fruitful. Future studies that focus on the molecular mechanisms of the T6SS effector and core proteins that impact plant pathogenesis will expand our understanding of the use of T6SSs in transkingdom interactions.
